# CARING FOR A VERY OLD PARENT WITH DEMENTIA: STRATEGIES EMPLOYED TO MANAGE BEHAVIORAL AND PSYCHOLOGICAL SYMPTOMS

**DOI:** 10.1093/geroni/igad104.0921

**Published:** 2023-12-21

**Authors:** Elizabeth Gallagher, Kathrin Boerner, Yijung Kim, Kyungmin Kim, Daniela Jopp

**Affiliations:** University of Massachusetts Boston, Boston, Massachusetts, United States; University of Massachusetts Boston, Boston, Massachusetts, United States; The University of Texas at Austin, Austin, Texas, United States; Seoul National University, Seoul, Republic of Korea; University of Lausanne, Lausanne, Vaud, Switzerland

## Abstract

Population aging has given rise to an increasingly common new group of caregivers, older adult children caring for their very old parents. These caregivers often find themselves balancing several other roles and responsibilities on top of caring for their parent, such as navigating their own health issues, caring for grandchildren, or transitioning into retirement. When a parent has dementia, this presents an additional challenge to caregivers, especially given that most persons with dementia exhibit behavioral and psychological symptoms of dementia (BPSD). The goal of the present research was to examine the range of strategies older caregivers employ in their daily lives in response to parents’ BPSD, using applied thematic analysis. Specifically, we sought to identify and describe the different caregiver strategies for managing BPSD and examine how these strategies are connected, including whether certain strategies co-occur. We conducted in-depth interviews with 100 caregivers (aged 65+) caring for parents with dementia (aged 90+) as part of the Boston Aging Together Study. Results revealed seven strategies used by child caregivers in managing BPSD—including “setting the record straight,” “going along with it,” distracting and redirecting, ignoring, telling “fiblets” or lies, medicating their parent to alleviate symptoms, and altering their physical environment. The most frequently co-occurring strategies employed by child caregivers included telling “fiblets” or lies as well as distracting and redirecting. This study has the potential to inform future caregiver interventions aimed at effectively addressing BPSD and improving quality of life for older child caregivers and their very old parents with dementia.

